# Characterization of the complete mitogenome of Haifa grouper, *Hyporthodus haifensis* (Perciformes: Serranidae), and its phylogenetic position within Epinephelini

**DOI:** 10.1080/23802359.2021.1904797

**Published:** 2021-03-31

**Authors:** Noel Vella, Adriana Vella

**Affiliations:** ^a^Department of Biology, Conservation Biology Research Group, University of Malta, Msida, Malta

**Keywords:** Mitochondrial genome, phylogeny, *Hyporthodus haifensis*, Epinephelini

## Abstract

The complete mitochondrial genome of the Haifa grouper, *Hyporthodus haifensis* (Ben-Tuvia, 1953), has been obtained, through Illumina next-generation sequencing, and annotated. This mitogenome was found to be 16,525 bp long and to contain 37 genes, a control region, and the L-strand replication origin. The overall base composition of the complete mitogenome for this species was found to be 28.55% A, 28.07% C, 16.32% G, and 27.06% T. This study also looked into the mitogenome phylogenetic relationships of *H. haifensis* within the tribe Epinephelini and adds to the genetic resources currently available for the species.

The Haifa grouper, *Hyporthodus haifensis* (Ben-Tuvia, 1953), belongs to the family Serranidae, subfamily Epinephelinae, tribe Epinephelini. The latter is composed of 16 genera and 171 species, with the genus *Hyporthodus* hosting 18 species (Heemstra and Randall 1993; Parenti and Randall 2020). Epinephelini species are renowned for their economic value and form an integral part of coastal fisheries also in the Mediterranean Sea (Vella and Vella 2016). The life-history of most Epinephelini makes these species vulnerable to overfishing (Sadovy 1994; Sadovy de Mitcheson et al. 2013; Giglio et al. 2017), with a number of them being listed in high-risk categories by IUCN (2020). Some Epinephelini species, such as *Epinephelus marginatus*, are considered as flagship species (Buchholz-Sørensen and Vella 2016) thus frequently studied, while others, such as *H. haifensis*, are rarely considered and are occasionally misidentified at fisheries landing sites with other groupers, such as *Mycteroperca rubra*, *E. marginatus* and *Epinephelus caninus* or with other species from unrelated taxa, such as *Polyprion americanum* (Vella and Vella 2016; *pers. obs*.). The latest IUCN assessment listed *H. haifensis* as Least Concern from its previous Data Deficient status (Francour and Pollard 2018), though the same report indicates that the species is naturally rare and due to the lack of life-history information should be closely monitored.

A 14.8 kg *H. haifensis* specimen was caught in February 2017 by local artisanal fishermen off the coast of Malta (35°45′N, 14°17′E). A tissue sample was collected from this specimen and deposited at the Ichthyological Collection of the Conservation Biology Research Group, University of Malta (www.um.edu.mt, Adriana Vella, adriana.vella@um.edu.mt) under the voucher number CBRG 170202019. The genomic DNA was extracted from the tissue sample using GF-1 DNA Extraction Kit (Vivantis Technologies, Subang Jaya, Malaysia), and a DNA library of the whole genome was constructed. Next-generation sequencing was used to sequence 2 × 150 bp end reads through Illumina HiSeqX (Illumina, San Diego, CA). Sequences were paired, trimmed at Q ≥ 30 and the complete circular mitogenome was de novo assembled using Geneious R10 (Kearse et al. 2012). The *tRNA* genes were identified through secondary structures using tRNAscan-SE version 2.0 (Chan and Lowe 2019), while protein-coding genes (PCGs), *rRNA* genes and the control region were identified through homology with other Epinephlini species (Zhuang et al. 2013).

The complete mitogenome for *H. haifensis* is 16,525 bp long (MW015093), falling in the range of other mitogenomes of Epinephelini species, which range between 16,389 bp in the Striped grouper, *Epinephelus latifasciatus* (KC480177, Lai et al. 2013) to 17,227 bp in the Duskytail grouper, *Epinephelus bleekeri* (KF556648, Wu et al. 2015). The mitogenome studied here contains 13 PCGs, two *rRNA* genes, 22 *tRNA* genes, and two non-coding regions (control region and O_L_) and follows the typical gene order of fish species (Satoh et al. 2016). The majority of the genes are encoded on the H-strand, except ND6 gene, eight *tRNA* genes, and O_L_. The PCGs lengths range between 168 bp (ATP8) and 1839 bp (ND5). Most of the PCGs utilize ATG as their start codon except COX1 which uses GTG similar to most other fish species (Satoh et al. 2016), and ATP6 which uses CTG. In general, the latter start codon for ATP6 is considered as unusual in fish species (Satoh et al. 2016), however, it was found to occur in 82% of the Epinephelini species included in Figure 1. Almost all genes have TAA as their stop codon, except ND3 that uses TAG, while COX2 and ND4 use the incomplete stop codon T–. The length of the 22 *tRNA* genes varies between 67 bp (Cys) to 77 bp (Leu^UUR^), and all produced the expected cloverleaf structure except for Ser^AGY^ that has the DHU arm missing.

**Figure 1.**
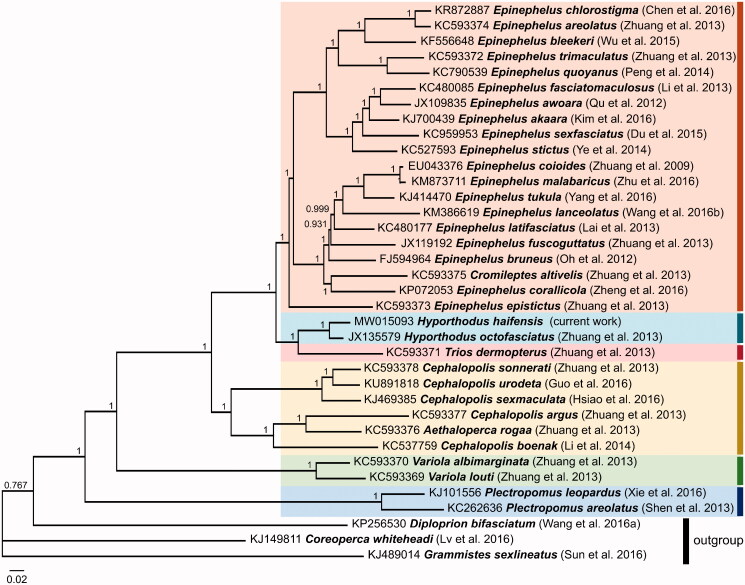
Bayesian Inference based phylogeny depicting the mitogenomic relationship (excluding the control region) between 33 Epinephelini species using other Serranidae species as outgroup. Each label includes the GenBank accession number, species, and reference (listed in the Supplementary Material). The numbers at the nodes indicate the posterior probability values. This analysis used 5 × 10^6^ generations, a sample frequency every 1,000 generations and a burn-in of 25%.

The mitogenome of *H. haifensis* was aligned with that of other Epinephelini species using ClustalW (Thompson et al. 1994), and a phylogenetic tree was constructed using Bayesian Inference analysis through MrBayes version 3.2.6 (Huelsenbeck and Ronquist 2001) (Figure 1) using GTR G + I substitution model as determined by jModelTest version 2.1.7 (Darriba et al. 2012). This analysis placed *H. haifensis* on the same branch as *Hyporthodus octofasciatus*, forming a sister group to *Triso dermopterus*. All species grouped together according to their respective genera, except for the monotypic genus *Cromileptes* (represented by *C. altivelis*) which nested within the genus *Epinephelus* and the monotypic genus *Aethaloperca* (represented by *A. rogaa*) which nested within the genus *Cephalopholis*. The position of these two monotypic genera is consistent with observations noted using other genetic markers (Craig and Hastings 2007) and other smaller data sets of Epinephelini mitogenomes (Zhuang et al. 2013). This outcome further supports the need for a taxonomic revision of these monotypic taxa within their respective clades as indicated in Zhuang et al. (2013). This study adds to the genetic resources available for *H. haifensis*, which can be used as a tool to promote further research and effective conservation of this rare species.

## Supplementary Material

Supplemental Material

## Data Availability

The genome sequence data that support the findings of this study are openly available in GenBank of NCBI at https://www.ncbi.nlm.nih.gov under the accession no. MW015093. The associated BioProject number is PRJNA661720.
