# Supplementary material for: Characterization of the complete mitogenome of Haifa grouper, Hyporthodus haifensis (Perciformes: Serranidae), and its phylogenetic position within Epinephelini
Source: Mitochondrial DNA B Resour. 2021 Mar 31;6(4):1287–9. doi: 10.1080/23802359.2021.1904797 (PMC8018344; doi:10.1080/23802359.2021.1904797)
Supplement: Supplemental Material [file TMDN_A_1904797_SM4769.docx]

**Supplementary Material:**

**References to the publications reporting the GenBank accession numbers of mitogenomes used for Figure 1.**

Chen X, Chen H, Yang W, Lin L, Liu M. 2016. Complete mitochondrial genome and the phylogenetic position of the brown-spotted grouper *Epinephelus chlorostigma* (Perciformes: Epinephelidae), Mitochondrial DNA Part A. 27(6):4268-4269.

Du FY, Ye L, Wang XH. 2015. Complete mitochondrial genome of the sixbar grouper *Epinephelus* *sexfasciatus* (Perciformes: Epinephelidae). Mitochondrial DNA. 26(3):461-462.

Guo M, Huang H, Gao Y. 2016. Complete mitochondrial genome of darkfin hind *Cephalopholis urodeta* (Perciformes, Epinephelidae), Mitochondrial DNA Part B. 1(1):913-916.

Hsiao ST, Chen KS, Tseng CT, Wu CL. 2016. Complete mitochondrial genome of the sixblotch hind *Cephalopholis sexmaculata* (Pisces: Perciformes). Mitochondrial DNA Part A. 27(2):1018-1019.

Kim YK, Lee YD, Oh HS, Han SH. 2016. The complete mitochondrial genome and phylogenetic position of the Endangered red-spotted grouper *Epinephelus akaara* (Perciformes, Serranidae) collected in South Korea, Mitochondrial DNA Part B. 1(1):927-928.

Lai T, He B, Peng Z, Wang X, Pan L. 2013. Complete mitochondrial genome of the striped grouper *Epinephelus latifasciatus* (Serranidae, Epinephelinae). Mitochondrial DNA. 24(5):510-512.

Li JL, Liu M, Wang YY. 2013. Complete mitochondrial genome of the rock grouper *Epinephelus fasciatomaculosus* (Pisces: Perciformes). Mitochondrial DNA. 24(6):625-626.

Li JL, Liu M, Wang YY. 2014. Complete mitochondrial genome of the chocolate hind *Cephalopholis boenak* (Pisces: Perciformes). Mitochondrial DNA*.* 25(3):167-168.

Lv L, Tian C, Liang X, Yuan Y, Zhao C, Song Y. 2016. The complete mitochondrial genome sequence of *Coreoperca whiteheadi* (Perciformes: Serranidae). Mitochondrial DNA Part A. 27(1):301-303.

Oh BS, Oh DJ, Jung MM, Jung YH. 2012. Complete mitochondrial genome of the longtooth grouper *Epinephelus bruneus* (Perciformes, Serranidae). Mitochondrial DNA. 23(2):137-138.

Peng Z, Chen J, Lai T, Huang Y, Wu L. 2014. Complete mitochondrial genome of the longfin grouper *Epinephelus quoyanus* (Serranidae: Epinephelinae). Mitochondrial DNA. 25(3):175-176.

Qu M, Zhang X, Ding SX. 2012. Complete mitochondrial genome of yellow grouper *Epinephelus awoara* (Perciformes, Epinephelidae). Mitochondrial DNA. 23(6):432-434.

Shen M, Shi X, Qu M, Chen J. 2013. Complete mitochondrial genome of squaretail coralgrouper *Plectropomus areolatus* (Perciformes, Epinephelidae). Mitochondrial DNA. 24(4):365-367.

Sun Y, Wei T, Su X, Li T. 2016. The complete mitochondrial genome of *Grammistes sexlineatus* (Perciformes, Serranidae). Mitochondrial DNA Part A. 27(2):821-823.

Wang H, Guo L, Ding S. 2016a. The complete mitochondrial genome of *Diploprion bifasciatum* (Perciformes, Serranidae), Mitochondrial DNA Part A, 27(5):3137-3138.

Wang X, Wang Q, Xie Z, He J, Wang D, Chen H, Li S, Zhang Y, Lin H. 2016b. The complete mitochondrial genome of the *Epinephelus lanceolatus* (Perciformes: Serranidae). Mitochondrial DNA Part A. 27(3):1738-1739.

Wu X, Xie Z, Yang L, Yang H, Yue L, Hou L, Zhang Y, Shu H. 2015. The complete mitochondrial genome of the duskytail grouper *Epinephelus bleekeri* (Serranidae: Epinephelinae). Mitochondrial DNA. 26(5):722-723.

Xie Z, Yu C, Guo L, Li M, Yong Z, Liu X, Meng Z. 2016. Ion Torrent next-generation sequencing reveals the complete mitochondrial genome of black and reddish morphs of the Coral Trout *Plectropomus leopardus*. Mitochondrial DNA Part A. 27(1):609-612.

Yang Y, Xie Z, Peng C, Wang J, Li S, Zhang Y, Zhang H, Lin H. 2016. The complete mitochondrial genome of the *Epinephelus tukula* (Perciformes, Serranidae). Mitochondrial DNA Par A. 27(1):520-522.

Ye L, Du FY, Wang XH, Wang Y. 2014. Complete mitochondrial genome of the black-dotted grouper *Epinephelus stictus*. Mitochondrial DNA. 25(2):87-88.

Zheng L, Xie J, Xie Z, Xiao L, Wang Q, Huang M, Chen H, Li S, Huang Z, Zhang Y. 2016. The complete mitochondrial genome of the *Epinephelus corallicola* (Perciformes: Serranidae). Mitochondrial DNA Part A. 27(6):3971-3972.

Zhu K, Huang G, Zhang D, Guo Y, Yu D. 2016. The complete nucleotide sequence of Malabar grouper (*Epinephelus malabaricus*) mitochondrial genome. Mitochondrial DNA Part A. 27(3):2087-2088.

Zhuang X, Ding S, Wang J, Wang Y, Su Y. 2009. A set of 16 consensus primer pairs amplifying the complete mitochondrial genomes of orange-spotted grouper (*Epinephelus coioides*) and Hong Kong grouper (*Epinephelus akaara*). Mol Ecol Resour. 9(6):1551-1553.

Zhuang X, Qu M, Zhang X, Ding S. 2013. A Comprehensive Description and Evolutionary Analysis of 22 Grouper (Perciformes, Epinephelidae) Mitochondrial Genomes with Emphasis on Two Novel Genome Organizations. PLoS One. 8(8):e73561.
